# Cenegermin for Treating Neurotrophic Keratitis: An Evidence Review Group Perspective of a NICE Single Technology Appraisal

**DOI:** 10.1007/s41669-019-0138-z

**Published:** 2019-06-25

**Authors:** Nigel Fleeman, James Mahon, Sarah Nevitt, Rui Duarte, Angela Boland, Eleanor Kotas, Yenal Dundar, Joanne McEntee, Sajjad Ahmad

**Affiliations:** 1 Liverpool Reviews and Implementation Group, Whelan Building, University of Liverpool, Liverpool, UK; 2 Coldingham Analytical Services, Berwick-upon-Tweed, UK; 3 North West Medicines Information Centre, Liverpool, UK; 4 Moorfields Eye Hospital NHS Foundation Trust, London, UK

## Abstract

As part of the Single Technology Appraisal (STA) process, the National Institute for Health and Care Excellence (NICE) invited the manufacturer of cenegermin (OXERVATE^®^, Dompé) to submit evidence for the clinical and cost effectiveness of cenegermin for neurotrophic keratitis (NK). The Liverpool Reviews and Implementation Group (LRiG) at the University of Liverpool was commissioned to act as the Evidence Review Group (ERG). This article summarises the ERG’s review of the evidence submitted by the company and provides a summary of the Appraisal Committee’s (AC) final decision. Clinical-effectiveness evidence from two phase II randomised controlled trials (RCTs) of cenegermin found cenegermin to improve corneal healing after 8 weeks compared with vehicle, considered a proxy for artificial tears. Longer-term data and comparisons with other relevant comparators were insufficient to draw conclusions. The company developed a de novo economic model that found cenegermin to be dominant when compared with artificial tears, except in one of seven scenarios. However, the ERG considered that the model had a major structural flaw in that it failed to allow patients to enter a ‘sustained healing’ state from ‘standard of care (SoC) non-healing’ and ‘SoC deteriorating’ states, or to move into an ‘SoC deteriorating’ state from an ‘SoC non-healing’ state. Following the first AC meeting, the company submitted a revised model with a revised model structure that removed the ‘SoC deteriorating’ state and introduced an ‘SoC healed’ state to sit alongside the existing ‘sustained healing’ and ‘SoC non-healing’ states from the original model. However, the ERG continued to express concerns, which included (1) extrapolation of the treatment effect of cenegermin over a patient’s lifetime; (2) the assumption that patients had two specialist visits a month; (3) the assumption that artificial tears, autologous serum eye drops and contact lenses continued for a lifetime after healing; (4) the simplified modelling of costs and utilities; and (5) the underlying uncertainty in the utility values. The ERG therefore considered the company’s model could not produce a robust incremental cost-effectiveness ratio (ICER) per quality-adjusted life-year (QALY) gained. The ERG did however present an alternative ICER by amending the use and cost of autologous serum eye drops, contact lenses and artificial tears in the ‘healed’ and ‘non-healed’ states. Applying these changes produced an ICER of £302,717 per QALY gained. Because of uncertainties with the clinical- and cost-effectiveness evidence, the AC concluded that cenegermin cannot be recommended within its marketing authorisation for NK.

## Key Points for Decision Makers

**Table:** 

Clinical-effectiveness evidence was presented from two trials (REPARO and Study 0214) that demonstrated cenegermin to improve corneal healing at 8 weeks when compared with vehicle, which was considered a proxy for artificial tears. There was however a lack of robust longer-term clinical-effectiveness data and data versus other relevant comparators for moderate to severe neurotrophic keratitis (NK).
The independent Evidence Review Group considered the economic model submitted by the company to be structurally flawed as it did not allow patients to enter a ‘sustained healing’ state from ‘standard of care (SoC) non-healing’ and ‘SoC deteriorating’ states or to move into an ‘SoC deteriorating’ state from an ‘SoC non-healing’ state.
The company produced a revised economic model but the ERG still considered this to be structurally flawed.
Due to uncertainties with the clinical- and cost-effectiveness evidence, the National Institute for Health and Care Excellence was unable to recommend cenegermin as a treatment option for moderate or severe NK.

## Introduction

The National Institute for Health and Care Excellence (NICE) is an independent organisation responsible for providing national guidance to the National Health Service (NHS) in England and Wales on a range of clinical and public health issues, including the appraisal of new health technologies. The NICE Single Technology Appraisal (STA) process is specifically designed for the appraisal of a single health technology for a single indication, where most of the relevant evidence lies with one manufacturer or sponsor and typically covers new technologies shortly after UK market authorisation is granted [1]. Within the STA process, the manufacturer or sponsor provides a written submission (alongside a decision analytic model) that summarises the estimate of the clinical and cost effectiveness of the technology. An external independent organisation (typically, an academic group) known as the Evidence Review Group (ERG), provides a critique of the company’s submission (the ERG report). Consultees, clinical specialists and patient representatives also provide additional information during the appraisal process.

Using a specification developed by NICE (the final scope), the NICE Appraisal Committee (AC) considers the company’s submission, the ERG report, and testimonies from experts and stakeholders in order to determine whether the technology represents a clinical- and cost-effective use of NHS resources. All stakeholders and the public have an opportunity to comment on the preliminary guidance issued by NICE in the form of an Appraisal Consultation Document (ACD), after which the AC meets again to produce the final guidance (Final Appraisal Determination [FAD]). The final guidance constitutes a legal obligation for NHS providers in England and Wales to provide a technology that is approved within its licensed indication [1].

This article presents a summary of the ERG report for the STA of cenegermin (OXERVATE^®^, Dompé, Milan, Italy) for treating neurotrophic keratitis (NK). The Liverpool Reviews and Implementation Group at the University of Liverpool was commissioned to act as the ERG for this STA. Full details of all relevant appraisal documents (including the appraisal scope, ERG report, company and consultee submissions, NICE guidance, and comments on each of these) can be found on the NICE website [2].

## The Decision Problem

NK is a disease of the cornea. Causes of NK include ocular herpes infection, chemical burns, long-term use of contact lenses, chronic use of topical eye medications, ablative treatment of trigeminal neuralgia and systemic conditions such as diabetes and multiple sclerosis [3, 4]. NK is usually unilateral (i.e. affecting one eye only) [3] and is characterised by reduced corneal sensitivity, spontaneous breakdown of the corneal epithelium and impairment of corneal healing [3, 4].

Epidemiological data on NK are sparse. The prevalence of NK has been estimated as 1.6/10,000 people, based on the prevalence of conditions that are associated with NK, namely keratitis after herpes infection (simplex or zoster) and after surgery for trigeminal neuralgia [4]. Given the rarity of the disease, NK has been classified by the European Medicines Agency (EMA) as an orphan disease [5].

The intervention considered in this appraisal was cenegermin, a non-surgical treatment, administered as eye drops six times daily over a course of 8 weeks. Cenegermin was granted marketing authorisation by the EMA on 6 July 2017 for the treatment of moderate (persistent epithelial defect) or severe (corneal ulcer) NK in adults [6]. The company estimated, from a survey of 12 clinical experts cited in its submission, that approximately half of the patients diagnosed with NK have stage 2 (moderate) or stage 3 (severe) NK.

In the decision problem described in the company submission (CS), the population to be addressed was identical to that specified in the NICE scope, which was also identical to the population for whom cenegermin is indicated [6]. The comparator to cenegermin specified in the NICE scope was established clinical management without cenegermin (which may include treatment of any underlying causes, preservative-free artificial tears, collagenase inhibitors, medical or surgical eyelid closure, serum eye drops, therapeutic contact lenses, and surgery). Collagenase inhibitors were considered by the company (and the ERG) to be an inappropriate comparator. This is because they should only be considered when stromal melting is present, but cenegermin is not indicated for use in patients who have stromal melting.

## Independent Evidence Review Group Report

The evidence provided by the company comprised an initial submission, an economic model (which is commercial in confidence) and the company’s response to the ERG’s clarification requests. The ERG report comprised a summary and critical review of the evidence for the clinical and cost effectiveness of the technology provided by the company. The role of the ERG was to:

- assess whether the evidence submitted by the company conforms to the methodological guidelines issued by NICE [7];
- assess whether the company’s interpretation and analyses of the evidence are appropriate;
- indicate the presence of other sources of evidence or alternative interpretations of the evidence that could help inform the development of NICE guidance.

In addition to providing this detailed critique, the ERG updated the company searches and modified a number of key assumptions and parameters within the company’s economic model to examine the impact of these changes on cost-effectiveness results. The ERG also critically reviewed evidence provided after publication of the ACD.

### Clinical Evidence

In July 2015, a systematic review was conducted by the company to identify published clinical trials of cenegermin and comparators for patients with NK. In August 2017, the company updated their search to identify evidence published since the original review was conducted.

The searches conducted by the company identified two phase II randomised controlled trials (RCTs) of cenegermin—REPARO [8] and Study 0214 [9]. Both trials were double-blinded, randomised, multicentre RCTs. Neither trial had been published in full in a peer-reviewed journal at the time of the appraisal, although limited data for REPARO had been presented in a conference abstract [10]. The results from REPARO have since been published in full, in September 2018 [11]. REPARO was a three-arm trial that included two cenegermin arms, each exploring different doses (10 µg/ml and 20 µg/ml). Both trials compared cenegermin with a vehicle arm. However, only data from the cenegermin 20 µg/ml arm were considered relevant to the appraisal since this is the licensed dose. This dose was also used in Study 0214. Vehicle was considered by the company to be a proxy for artificial tears. The company’s risk of bias assessment found both trials to be at low risk of bias.

Given that direct evidence was only available for cenegermin versus vehicle, the company also undertook a separate ‘clinical extension review’ to identify studies to inform a mixed treatment comparison (MTC) to compare cenegermin with all relevant comparators. Studies considered for inclusion in the MTC were the two cenegermin trials plus 23 studies of comparator treatments, of which only one study was an RCT [12]. In this trial, amniotic membrane transplantation (*n *=15) was compared with ‘conventional management’ (tarsorrhaphy [a surgical procedure in which the eyelids are sewn together], *n *=11; bandage contact lens, *n *=4).

The company considered that the results from its MTC were associated with such uncertainty that no conclusions could be drawn. Thus, the results were not reported in the CS (other than in an appendix, for transparency). Evidence was therefore presented narratively from REPARO and Study 0214. A meta-analysis of data from these two trials was also presented. The meta-analysis results had previously been reported at the Congress of the European Society of Ophthalmology in June 2017 [13].

Excluding 52 patients treated with the unlicensed cenegermin dose, REPARO included approximately twice as many patients relevant to the decision problem (*n *=104) as Study 0214 (*n *=48). In most respects, the study characteristics of the two cenegermin trials were very similar. One of the main differences was that REPARO was conducted in Europe and Study 0214 was conducted in the US. Another noteworthy difference was that both the cenegermin and vehicle formulations contained methionine as an excipient in Study 0214, unlike in REPARO. This is noteworthy because the licensed formulation of cenegermin also contains methionine (i.e. identical to that used in Study 0214).

Corneal healing was measured at 8 weeks in both trials; this was the primary outcome in Study 0214, while in REPARO, the primary outcome was corneal healing at 4 weeks. Corneal healing was predefined as the greatest diameter of the corneal fluorescein staining in the area of the persistent epithelial defects (PED) or corneal ulcer being < 0.5 mm. At the request of the US FDA, corneal healing was also defined post hoc as “no residual fluorescein staining in the area of the corneal lesion (0 mm) and no persistent staining elsewhere in the cornea,” where persistent staining was defined as “staining not changing in shape and/or location of different time points”. Within both trials, assessments of corneal healing were performed by assessors at a central reading centre who evaluated the clinical pictures of corneal fluorescein staining. These assessors were masked to the treatment arm from which the pictures of corneal fluorescein staining were derived.

In REPARO, 74.0% and 72.0% of patients treated with cenegermin achieved complete healing at 8 weeks (< 0.5 mm and 0 mm, respectively), and in Study 0214, 69.6% and 65.2% achieved complete healing at 8 weeks (< 0.5 mm and 0 mm, respectively). The difference in the percentage of patients achieving complete healing (< 0.5 mm) between the cenegermin and vehicle arms at 8 weeks was 30.9% (97.06% confidence interval [CI] 10.60–51.13; *p * = 0.002) in REPARO and 40.4% (95% CI 14.2–66.6; *p  *= 0.006) in Study 0214. The difference in the percentage of patients achieving complete healing (0 mm) between the cenegermin and vehicle arms at 8 weeks was 38.7% (97.06% CI 18.72–58.62; *p * < 0.001) in REPARO and 48.6% (95% CI 24.0–73.1; *p * < 0.001) in Study 0214.

The meta-analysis results were presented using both definitions of corneal healing. Both pooled odds ratios (ORs) were in favour of cenegermin versus vehicle. The meta-analysis of corneal healing to < 0.5 mm at 8 weeks provided a pooled OR of 4.24 (95% CI 2.11–8.50; *p * < 0.001), and the meta-analysis of corneal healing to 0 mm in the lesion area, with no persistent staining elsewhere at 8 weeks, provided a pooled OR of 6.09 (95% CI 2.97–12.50; *p  *< 0.001).

Other outcomes of the trials at 8 weeks included complete corneal clearing, visual acuity, corneal sensitivity, deterioration in NK, recurrence of NK, adverse events (AEs) of treatment and health-related quality of life (HRQoL). HRQoL was measured in both trials using the European Quality of Life-5 Dimensions Questionnaire (EQ-5D-5L), EQ-5D visual analytic scale (VAS) and National Eye Institute Visual Functioning Questionnaire 25 (NEI-VFQ-25). There were no significant differences between trial arms for any of these secondary outcomes. While AE frequencies differed between trials (45.2% in REPARO, 83% in Study 0214), the company reported that most AEs were mild or moderate in severity and did not require treatment discontinuation or any corrective treatment. AEs included eye disorders, with the most common type (as a proportion of all patients in each arm) being eye pain (9.6% and 7.7% in the cenegermin and vehicle arms of REPARO, respectively, and 30.4% and 8.3% in the cenegermin and vehicle arms of Study 0214, respectively). The results from the EQ-5D-5L analyses were highly variable, with no consistent pattern in terms of change from baseline to 8 weeks in either arm, for either trial. No statistically significant differences between arms from baseline to 8 weeks were found in either trial with regard to the mean change for EQ-5D VAS and NEI-VFQ-25. The company considered that no robust interpretations or conclusions could be drawn from the HRQoL results.

In addition to a controlled treatment period of 8 weeks, both trials included an uncontrolled 8-week cenegermin treatment period and an extended follow-up period (48 weeks in REPARO, 24 weeks in Study 0214). Following the 8-week controlled period, patients in the cenegermin arm who were completely healed, as well as patients who were not completely healed (but who had not deteriorated either), entered the extended follow-up period. Patients in the vehicle arm who had deteriorated during the 8-week controlled treatment period, as well as those who were not completely healed at the end of the 8-week controlled treatment period, entered the 8-week uncontrolled treatment period before entering the extended follow-up period. The only patients randomised to vehicle who entered the extended follow-up period without the need for an uncontrolled treatment period were patients who achieved corneal healing during the controlled treatment period.

Outcomes measured in the extended follow-up period were recurrence rates. Patients who were healed at the end of the controlled treatment period but no longer healed at the end of the extended follow-up period were considered to have had a recurrence of NK. Recurrence rates at the end of the extended follow-up were found to vary from 0 to 30% depending on the trial arm that patients were initially randomised to and whether complete healing had been achieved during the controlled or uncontrolled treatment periods. However, not all patients had a response available at the follow-up points, and healing rates were calculated in the set of patients for whom response data were available, rather than for the intention-to-treat (ITT) population. Therefore, the company considered these findings to be indicative only, precluding any firm conclusions to be drawn.

### Critique of the Clinical Evidence and Interpretation

Overall, the ERG considers that the methods used to conduct the clinical-effectiveness systematic review and the ‘clinical extension review’, as described in the CS, appeared to be satisfactory. The ERG also considered that the methodological approaches carried out by the company to conduct its MTC were appropriate. The ERG agreed with the company that the MTC data were limited and that the uncertainty associated with the results was so large that the results were difficult to interpret, precluding any firm conclusions from the data.

The ERG concurred with the company that the vehicle used in the two cenegermin trials was similar in its composition to artificial tears as it contained ingredients widely used in commercially available preservative-free artificial tears. While the ERG noted some differences in baseline characteristics within and across the two cenegermin trials, the ERG considered that the baseline characteristics of the patient populations in these trials appeared to be similar to those who would likely be treated with cenegermin in NHS clinical practice.

The ERG agreed with the company that the two trials were at low risk of bias. However, the ERG noted that withdrawal rates during the controlled treatment period were quite high and unbalanced across the treatment arms in both studies (25% in the cenegermin arm in both trials, 7.7% in the vehicle arm of REPARO and 37.5% in the vehicle arm of Study 0214).

The ERG noted that several analysis approaches had been considered to take account of these missing data, with the last observation carried forward (LOCF) method used within the primary analysis in both trials. The ERG considered that the LOCF method ignores the uncertainty introduced by missing outcome data and that the multiple imputation approach would better capture the uncertainty introduced by the missing outcome data as this is a more statistically powerful approach. The results of sensitivity analyses presented by the company, including the multiple imputation approach, were however similar to the results using the LOCF method.

Corneal healing was considered by the ERG to be the most appropriate outcome for measuring the efficacy of cenegermin, noting that it is a common outcome in studies of eye diseases. The ERG considered the efficacy data show that, after 8 weeks, cenegermin is superior to vehicle in terms of corneal healing, but noted that the reasons for the differences in AE frequencies across trials were unknown. The ERG concurred with the company that no robust interpretations or conclusions could be drawn from the HRQoL results.

The ERG considered that the evidence from the extended follow-up periods of both cenegermin trials suggested that a high proportion of patients who are healed after 8 weeks of treatment with cenegermin remain healed after a further 24 weeks (REPARO and Study 0214) and 48 weeks (REPARO). However, the ERG agreed with the company that it is difficult to draw firm conclusions from these exploratory analyses.

### Cost-Effectiveness Evidence

The company developed a de novo economic model in Microsoft Excel (Microsoft Corporation, Redmond, WA, USA) to compare the cost effectiveness of treatment with cenegermin with preservative-free artificial tears (using clinical-effectiveness data for vehicle as a proxy for artificial tears). The cost-effectiveness model presented by the company comprised two stages: a decision tree, followed by a Markov model. After the initial treatment period of 8 weeks, which determined the outcome associated with initial treatment, patients entered a Markov process with three NK states: ‘sustained healing’, ‘standard of care (SoC) non-healing’ or ‘SoC deteriorating’. ‘Death’ was an absorbing state. The company used a cycle length set to 4 weeks. The economic evaluation was undertaken from the perspective of the NHS, and the model time horizon in the base case was the lifetime of patients, set at 100 years of age for the cohort, with 5-, 10- and 20-year time horizons included as scenario analyses. Outcomes were measured in quality-adjusted life-years (QALYs) and both costs and QALYs were discounted at a rate of 3.5% per annum, as recommended by NICE. Cenegermin was costed at its list price and artificial tears from the British National Formulary at 2017 prices [14]. Resource use and treatment/surgical costs for the different health states were estimated based on the company’s survey of 12 clinical experts. Resource use/costs included visits to specialists and treatments provided in an SoC ‘basket’ (such as tarsorrhaphy, serum eye drops and contact lens). Unit costs used by the company were derived from official sources (UK National Reference Costs 2015/2016 [15]) and other sources [16, 17].

In the company base case, cenegermin was dominant when compared with artificial tears, generating more benefits (+ 0.08 QALYs) at a decreased cost of £21,549. The company carried out a range of deterministic sensitivity analyses. Varying the utility of the ‘SoC non-healing’ states in the follow-up model had the biggest effect on the company’s cost-effectiveness results, followed by the starting age, the discount rate and the probability of healing with cenegermin versus artificial tears, although cenegermin remained dominant or had an ICER below £20,000 per QALY gained.

The company’s probabilistic sensitivity analysis (PSA) involved varying only a limited number of parameters. The results of the company’s PSA suggested that there is a 97.6% probability of treatment with cenegermin being cost effective at a willingness to pay threshold of £20,000 per QALY gained, and a 97.7% probability of cenegermin being cost effective at a willingness to pay threshold of £30,000 per QALY gained.

The company carried out seven scenario analyses. The only scenario in which cenegermin was not dominant versus artificial tears was when considering a time horizon of 5 years. In this scenario, cenegermin generated more benefits than artificial tears (+ 0.02 QALYs) at an increased cost of £3139. The incremental cost-effectiveness ratio (ICER) for this scenario for the comparison of cenegermin versus artificial tears was £127,390 per QALY gained.

### Critique of the Cost-Effectiveness Evidence and Interpretation

The ERG did not consider the model produced by the company to be fit for purpose. In the company model, it was assumed that patients who do not achieve ‘sustained healing’ with initial treatment with cenegermin or artificial tears will never achieve ‘sustained healing’ and only had palliative treatments with frequent (up to 10 times per month) visits to specialists for the rest of their lives. The ERG considered this high number of visits to be implausible. The ERG also considered that the implicitly assumed zero efficacy associated with treatments in the SoC ‘basket’ at achieving ‘sustained healing’ contrasted with the results of the company’s own survey of clinical experts.

Following concerns raised by the ERG, the company suggested that lower estimates for the number of specialist visits for patients without ‘sustained healing’ would be appropriate. This change moved cenegermin from being dominant in the base case to having an ICER of £22,737 per QALY gained compared with artificial tears.

Even if the ERG had been satisfied with the model structure, there were errors in the way utility values and the costs of one-off treatments were applied in the model. Correcting these errors, together with the reduced number of specialist visits, resulted in an ICER of £128,453 per QALY gained. However, due to the model’s flawed structure, the ERG did not present this figure as a preferred ICER but as a more accurate estimate of the company base-case ICER within the confines of the flawed model structure. The ERG considered that this value was still likely to be an underestimate of the base-case ICER per QALY gained, for three main reasons: (1) the average number of specialist visits for people initially treated with artificial tears was implausibly high at approximately 450 over a patient’s lifetime; (2) while the utility decrements for tarsorrhaphy were uncertain, the values used by the company overestimated these decrements since all patients with tarsorrhaphy were assumed to suffer unilateral blindness from the procedure; and (3) mortality in the model was probably underestimated.

### Conclusions of the ERG Report

Clinical trial evidence from two phase II RCTs demonstrated that at 8 weeks, for patients with stage 2 or 3 NK, cenegermin resulted in improved corneal healing compared with vehicle. In the trials, vehicle was similar to artificial tears used in NHS clinical practice, and the population of patients had characteristics similar to those of patients seen in the NHS. However, the ERG highlighted that only 24 patients included in the trials were randomised to receive the commercially available formulation of cenegermin (i.e. including methionine) and only a further 10 patients received this formulation during an uncontrolled 8-week treatment period (all 34 patients were in Study 0214). Furthermore, while artificial tears are used to treat patients with stage 2 and 3 NK in clinical practice, they are often used in addition to other interventions. While the company conducted an MTC to compare cenegermin with other interventions, the results were associated with such uncertainty that no conclusions could be drawn from the results of the MTC.

The ERG considered that the company submitted a cost-effectiveness model that had a major structural flaw, namely the model failed to allow patients to enter a ‘sustained healing’ state from ‘SoC non-healing’ and ‘SoC deteriorating’ states or to move into an ‘SoC deteriorating’ state from an ‘SoC non-healing’ state. The ERG considered that this structural flaw resulted in such implausible resource use assumptions that the model was not fit for purpose. Without restructuring and reconstructing the model (which is beyond the remit of the ERG), the ERG could not present a plausible or preferred ICER per QALY gained. Due to the model’s structural flaw, the ERG considered a more accurate estimate of the company’s base-case ICER, within the confines of the flawed model structure, to be £128,453 per QALY gained.

## Key Methodological Issues

Two key methodological issues were highlighted by the ERG: (1) a lack of clinical-effectiveness evidence to enable a comparison of cenegermin with any other comparator other than vehicle (a proxy for artificial tears); and (2) the major structural flaw with the company’s model that did not allow patients to enter a ‘sustained healing’ state from ‘SoC non-healing’ and ‘SoC deteriorating’ states or to move into an ‘SoC deteriorating’ state from an ‘SoC non-healing’ state, rendering the model unfit for purpose. While the company also recognised the lack of clinical-effectiveness evidence, the company did not agree with the ERG that that the submitted model was structurally flawed. The company considered that the submitted model was simple but structurally robust and that the ICERs per QALY gained generated were informative.

Nonetheless, following the first AC meeting (ACM), the company did submit a revised model with a revised model structure. In modifying the original model, the company removed the ‘SoC deteriorating’ NK state and introduced an ‘SoC healed’ state to sit alongside the existing ‘sustained healing’ and ‘SoC non-healed’ states from the original model. This model allowed people having treatments from the SoC ‘basket’ to move from the ‘non-healing’ to ‘healed’ states. However, the ERG continued to express concerns with the assumptions used to inform the model as well as the model structure itself.

Regarding clinical effectiveness, the probability of ‘healing’ or ‘not healing’ for patients in the SoC ‘basket’ used in the revised model was taken from the company’s survey of 12 clinical experts. However, the responses varied widely in the experts’ estimates of effectiveness. Evidence from the survey incorporated into the model suggested that all treatments, other than autologous serum eye drops and contact lenses, had at least 72% efficacy, similar efficacy to that assumed for cenegermin in the model. It is noteworthy that, in the survey, the efficacy of artificial tears was found to be 77%, which is higher than the 38% used in the model. This means that either several of the current treatments for NK are more efficacious than cenegermin (in which case, given their lower cost, several treatments would dominate cenegermin), or the estimates from the survey were incorrect (which would render the results from the model unreliable). The ERG also noted that the probability of complete healing of the SoC ‘basket’ was taken as the weighted average of the proportion of people receiving each treatment (according to the survey of clinical experts) and the probability that each treatment achieved complete healing. As a person could receive more than one treatment, this means that it was mathematically possible that the probability of complete healing with the SoC ‘basket’ could have been greater than one.

The ERG identified a number of other issues with the revised model. These included the following assumptions, which were not supported by evidence and/or seemed to be clinically implausible.

- As in the original model, the treatment effect of cenegermin was extrapolated over a patient’s lifetime.
- As in the original model, it was assumed that patients had two specialist visits per month, which equates to 24 specialist visits per year, or around 450 over a lifetime.
- As in the original model, the use of artificial tears, autologous serum eye drops and contact lenses were assumed to continue for a lifetime after healing.

Furthermore, the ERG also highlighted the following.

- In the revised model, people with recurrence between cycles 1 and 13 (year 1) had different costs and utilities to those with recurrence after cycle 14 (because there were no surgical treatments after 1 year). The ERG considered that in order to produce an accurate ICER for decision making, recurrence costs and utilities would need to be modelled using a more sophisticated approach.
- The only difference between the ‘sustained healing’ and ‘SoC non-healing’ states in the company’s original model was a disutility applied for tarsorrhaphy. In the revised model, the disutility for tarsorrhaphy occurred in all health states in the first year only. Although this addressed the inaccuracy in how utility values were previously applied, it did not address the underlying uncertainty in the utility values themselves. Because of this, the QALY gain for cenegermin would likely be overestimated.

Given the structural flaws identified by the ERG, as with the company’s original model, without restructuring and reconstructing the model, the ERG could not present a plausible or preferred ICER per QALY gained. However, the ERG presented an alternative ICER per QALY gained by making the following amendments: (1) autologous serum eye drops and contact lens use stopped after 1 year in the healed state; (2) use of artificial tears stopped after 1 year in the ‘sustained healing’ and ‘healed’ states; and (3) all costs assumed to be the same in the ‘healed’ and ‘non-healed’ states in the first year. Taken together, applying these changes produced an ICER per QALY gained of £302,717.

## National Institute for Health and Care Excellence Guidance

At the first ACM, the AC was minded not to recommend cenegermin for treating NK. Following the company’s response to the ACD and their submission of a revised model, a second AC meeting was held. Following the second AC meeting, the AC concluded that cenegermin cannot be recommended within its marketing authorisation to treat moderate or severe NK. The key issues arising from the two meetings are summarised below.

### Consideration of Current Treatment Options

The clinical experts highlighted that current treatments are palliative in nature and that there is no standard care pathway for patients with NK. The choice of treatment depends on the severity of the disease, clinician preference, patient need, and availability. Autologous serum eye drops, which are not available in many centres, may take 6–8 weeks to prepare. Tarsorrhaphy is considered to be relatively cheap and effective, but it is not a popular treatment option with patients because it can cause disfigurement. The AC agreed with the clinical experts that people with NK have an unmet clinical need and would welcome any new treatment that improves outcome and reduces the need for surgery. The AC concluded that, if available, cenegermin would be used as a potential early option and topical treatments may be used concomitantly.

### Consideration of the Clinical-Effectiveness Issues

Clinical experts consulted at the first ACM agreed treatment in the vehicle arm was not a placebo treatment because it would have some therapeutic benefit, akin to that of artificial tears. It was therefore not unreasonable to assume vehicle to be a proxy for artificial tears. While the AC considered the REPARO and Study 0214 trial populations to be generalisable to clinical practice in England, there were four limitations associated with the two trials: low patient numbers (as expected considering the rarity of the disease); limited long-term follow-up data; high withdrawal rates; and only a small number of patients received the licensed methionine-containing formulation of cenegermin (*n *=34). The AC therefore concluded that although cenegermin is more clinically effective than vehicle at 8 weeks, the results of both trials were associated with significant uncertainty, particularly after 8 weeks.

### Consideration of the Cost-Effectiveness Issues

The AC considered the company’s original model to be structurally flawed and its results not robust. The AC also noted anomalies in the cost-effectiveness results using the revised model, and questioned the reliability of the model results. While acknowledging the challenges of modelling a complex disease area with no established treatment pathway and minimal clinical evidence, these limitations needed to be accounted for in its decision making.

The AC therefore concluded that the benefits for cenegermin modelled by the company were likely to be an overestimate. In addition, uncertainty regarding the longer-term corneal healing effects of cenegermin meant it was not possible to identify a robust cost-effectiveness estimate for cenegermin versus artificial tears. While recognising that the company considered cenegermin to be innovative because it is the only treatment that has shown a relative improvement in corneal healing for treating NK, the AC concluded that it had not been presented with evidence of any additional benefits that were not captured in the QALY calculations. Based on the evidence presented, the AC considered the most likely ICER for cenegermin compared with artificial tears would be higher than the range that NICE normally considers to be an acceptable use of NHS resources (£20,000–£30,000 per QALY gained).

### Final Guidance

NICE guidance was issued on 18 July 2018 [2]. Because of uncertainties with the clinical- and cost-effectiveness evidence, the AC concluded that cenegermin cannot be recommended within its marketing authorisation to treat moderate or severe NK.

## Conclusions

The ERG considered that the clinical-effectiveness evidence from REPARO and Study 0214 demonstrated cenegermin to be superior to vehicle (considered a proxy for artificial tears) over 8 weeks. However, there is no evidence of a long-term benefit of cenegermin or of evidence supporting its clinical effectiveness versus other treatments used to manage moderate or severe NK. The AC agreed with these conclusions reached by the ERG. In addition, given the structural flaws, identified by the ERG, in the company’s model used to estimate cost effectiveness, the AC was unable to recommend cenegermin as an NHS treatment option for patients with moderate or severe NK in England. It is unclear if a different conclusion regarding the cost effectiveness could have been reached if a more sophisticated approach had been employed in the model, given the limitations with the clinical-effectiveness evidence.
